# Study protocol for the Maule Cohort (MAUCO) of chronic diseases, Chile 2014–2024

**DOI:** 10.1186/s12889-015-2454-2

**Published:** 2016-02-04

**Authors:** Catterina Ferreccio, Juan Carlos Roa, Claudia Bambs, Alejandra Vives, Alejandro H. Corvalán, Sandra Cortés, Claudia Foerster, Johanna Acevedo, Andrea Huidobro, Alvaro Passi, Pablo Toro, Yerko Covacevich, Rolando de la Cruz, Jill Koshiol, Mauricio Olivares, Juan Francisco Miquel, Francisco Cruz, Raúl Silva, Andrew F. Quest, Marcelo J. Kogan, Pablo F. Castro, Sergio Lavandero

**Affiliations:** 1 Advanced Center for Chronic Diseases (ACCDiS), Facultad Medicina, Pontificia Universidad Católica de Chile, Santiago, Chile; 2 Facultad Medicina, Universidad Católica del Maule, Talca, Chile; 3 Division of Cancer Epidemiology and Genetics, Infections and Immunoepidemiology Branch, National Cancer Institute, Bethesda, MD USA; 4 Hospital Santa Rosa de Molina, Curicó, Chile; 5 Advanced Center for Chronic Diseases (ACCDiS), Facultad Medicina, Universidad de Chile, Santiago, Chile; 6 Advanced Center for Chronic Diseases (ACCDiS), Facultad Ciencias Químicas y Farmacéuticas, Universidad de Chile, Santiago, Chile; 7 Cardiology Division, Department of Internal Medicine, University of Texas Southwestern Medical Center, Dallas, TX USA

**Keywords:** Prospective studies, Cohort studies, Population surveillance, Chronic disease/epidemiology, Agricultural workers diseases, Cardiovascular diseases, Neoplasms/Epidemiology

## Abstract

**Background:**

Maule Cohort (MAUCO), a Chilean cohort study, seeks to analyze the natural history of chronic diseases in the agricultural county of Molina (40,000 inhabitants) in the Maule Region, Chile. Molina´s population is of particular interest because in the last few decades it changed from being undernourished to suffering excess caloric intake, and it currently has the highest national rates of cardiovascular diseases, stomach cancer and gallbladder cancer. Between 2009 and 2011 Molina´s poverty rate dropped from 24.1 % to 13.5 % (national average 20.4 %); in this period the county went from insufficient to almost complete basic sanitation. Despite these advances, chemical pollutants in the food and air are increasing. Thus, in Molina risk factors typical of both under-developed and developed countries coexist, generating a unique profile associated with inflammation, oxidative stress and chronic diseases.

**Methods/Design:**

MAUCO is the core project of the recently established Advanced Center for Chronic Diseases (ACCDiS), Universidad de Chile & Pontificia Universidad Católica de Chile. In this study, we are enrolling and following 10,000 adults aged 38 to 74 years over 10 years. All eligible Molina residents will be enrolled. Participants were identified through a household census. Consenting individuals answer an epidemiological survey exploring risk factors (psycho-social, pesticides, diet, alcohol, and physical activity), medical history and physical and cognitive conditions; provide fasting blood, urine, and saliva samples; receive an electrocardiogram, abdominal ultrasound and bio-impedance test; and take a hand-grip strength test. These subjects will be re-interviewed after 2, 5 and 7 years. Active surveillance of health events is in place throughout the regional healthcare system. The MAUCO Bio-Bank will store 30 to 50 aliquots per subject using an NIH/NCI biorepository system for secure and anonymous linkage of samples with data.

**Discussion:**

MAUCO´s results will help design public health interventions tailored to agricultural populations in Latin America.

## Background

In just 40 years, the Chilean population experienced major socio-environmental changes associated with significant shifts in its health profile. Between 1970 and 2010, life expectancy increased from 64.7 to 81.3 among women and from 58.5 to 75.8 years among men. On average, life expectancy increased by 7 months per year between 1970 and 1990, and 3 months per year between 1990 and 2010 [1]. Unfortunately, for most of the population these additional years are affected by chronic diseases (CDs). Cross-sectional health surveys of the Chilean population in 2003 [2] and 2009 [3] revealed high and generally increasing prevalence of CDs and their risk factors: hypertension (32.3 % and 29.2 % in 2003 and 2009, respectively), diabetes (6.3% and 9.4%), high risk of cardiovascular disease on the Framingham score (14.0 % and 17.7 %), overweight (37.8% and 39.3 %), obesity (23.2 % and 25.1 %), high cholesterol (35.4 % and 38.5 %) and tobacco consumption (42.0 % and 41.0 %). Given that the prevalence of risk factors is still increasing, the peak of CD burden is yet to come.

In contrast to the health transition of most developed countries, in Chile the increase in life expectancy occurred before most of the population achieved socioeconomic development. In fact, a large proportion of the population is still living in poverty (20.4 %) [4]. The increase in population survival is the result of the high coverage of maternal and child healthcare, immunization and food supplementation programs, and a decline in fertility rates [5]. Simultaneously, a centralized national sanitation policy secured clean drinking water for the whole country. Even though coverage of safe drinking water grew rapidly in the 1970s and reached 97 % of population by 1990, there was virtually no wastewater treatment in 1990. Thus, while people drank clean water, they ate food that was irrigated with highly contaminated wastewater [6]. Since then, coverage increased substantially and currently includes 98% of the population [7]. Besides sanitation, very little has been done to control pesticides and chemicals in food throughout the country. Similarly, air pollution is increasing rapidly in large and small cities, and in the latter is largely, but not exclusively, associated with wood-based heating. The Chilean regulatory system is more permissive than the WHO guidelines [8, 9]. Thus, in Chile, risk factors for CD development cohabitate with the remains of underdevelopment [5], including a high burden of chronic infections (*H. pylori* >73 %) [10], together with high rates of obesity [3]. Little is known about the natural history of CDs in this particular socio-environmental context, and it remains unclear which are the most appropriate health risk indicators and/or prevention strategies.

The general objectives of the study are: i) To analyze the natural history of CDs in a high risk population by measuring the prevalence and evolution of CDs and their risk factors. The health outcomes of interest include: cardiovascular disease, metabolic syndrome, cancer, obesity, aging, mental health, quality of life, oral health and respiratory diseases. The risk factors of interest include: socio-economic, occupational, psychological, lifestyle (diet, physical activity, tobacco, and alcohol consumption), environmental, genetic and ethnic exposures. ii) To build a capacity to develop advanced epidemiological, clinical and basic research in CDs in Chile and specifically to create Data-Bank and Bio-Banks with an appropriate infrastructure to facilitate access to the data and samples for studies that address relevant research questions.

Our aim is to establish the first Chilean population cohort of CDs in the town of Molina in the Maule region, the Maule Cohort (MAUCO). This region in Central Chile has the highest standardized national mortality rates (SMR) for CDs [11], with 46.5 % of the population residing in the highest quintile of mortality for cardiovascular disease and 61 % for cerebrovascular ischemic disease [11], as well as the highest incidence of stomach cancer [10, 12]. Within the Maule region, Molina County has the highest SMR due to diabetes, dementia, Alzheimer’s disease, cardiovascular diseases and pneumonia. Males have excessively increased risk of mortality due to cirrhosis, stomach and prostatic cancer and heart ischemic illnesses, while woman are afflicted by gallbladder cancer, hypertension and non-ischemic heart diseases [13]. We hypothesize that common environmental factors are at the origin of these chronic conditions possibly causing chronic inflammation and oxidative stress.

MAUCO is the core project of the new Advanced Center for Chronic Diseases (ACCDiS), supported by the Fund for Research Centers in Priority Areas Program (FONDAP) of the Chilean National Commission for Scientific and Technological Research (CONICYT). MAUCO is initially funded for 10 years, and in this period MAUCO will recruit 10,000 adults from the general population and conduct follow-up visits.

## Methods/Design

### Study design and setting

MAUCO is a prospective population-based cohort study of individuals aged 38 to 74 years.

The Maule Region is one of the 15 administrative divisions of Chile, and the capital is Talca. This region has a surface area of 20,600 km^2^ with 955,048 inhabitants. Molina, one of its 30 counties, is 1,552 km^2^ in size (599 sq. mi) and lies 200 km south of Santiago, latitude -35.1165, longitude -71.2830 and altitude of 243 m above sea level [14]. Molina has 42,859 inhabitants, 30.1 % living in rural areas and 50.2 % male. In 2009, the poverty rate was 24.1% (CI = 17.7-30.2 %), and it dropped to 13.5 % (CI = 9.3-17.3 %) in 2011, representing a 40 % reduction in only 2 years. These changes were largely due to agricultural modernization and position Molina well above the national average (24.3 % of population living in poverty in 2011) [4, 15]. In addition, 87 % of the Molina population benefit from access to the public health system [16].

### MAUCO’s organization

a) Directorate: The cohort has an Epidemiologist Director (C.F.), an Executive Committee, comprised of the six ACCDiS principal investigators (S.L., C.F., P.F.C, A.F.Q, A.C., M.J.K.) and an Advisory Board. b) Advisory Board: The Advisory Board includes representatives of the Ministry of Health, the Regional Health Service, the Funding Agency, non-profit health organizations, Scientific Societies and representatives of the community. The Board defines the criteria and the decision-making processes regarding the use and sharing of data and samples. c) Field team: The field team includes epidemiologists, nurses, medical technologists, healthcare assistants and administrative clerks who 1) conduct the census, enrollment, survey and examination of participants and 2) collect samples, enter data and communicate with the community. d) Health surveillance team: The surveillance team is composed of clinicians, nurses, laboratory technologists and healthcare assistants, who actively search for events affecting the cohort participants in any of the region’s public hospitals. e) MAUCO epidemiologists and data managers: Led by the MAUCO Director, this team is responsible for the design, implementation, quality and accuracy of the study, data synthesis and analysis.

### Participants and selection criteria

All Molina residents aged 38 to 74 years are potentially eligible. To be eligible, individuals must have resided in Molina for at least 6 months of the past year and do not plan to move from Molina within 3 years of enrollment. Individuals who cannot give informed consent autonomously (e.g., cognitive impairment, dementia) or have a terminal illness are excluded.

We identify the geographic distribution of eligible residents through a household census, which records their age and sex, as well as basic household data (water and electricity provision, housing construction materials) and coordinates (latitude-longitude coordinate’s format, WGS84). Google Maps and Open Street Maps are used for geo-referencing. Based on census estimates [16], we computed a target population of 14,000 individuals, with an expected response of 80 % participation at baseline (11,200), 90 % of which would be finally eligible (10,800). Thus, the goal for enrollment was set at 10,000 participants.

### Recruitment

Individuals are contacted at their homes where the objectives of the study are explained in a brochure and the consent form. Accepting participants receive the first interview and examination in their homes. This first assessment consists of a 90 min health and risk factor questionnaire validated for the Chilean population (Table 1), physical condition evaluation, blood pressure and heart rate measurements and cognitive tests (Table 2). They are then appointed to the MAUCO health-station, located alongside the public hospital of Molina, to complete their baseline evaluation. At the MAUCO health-station, fasting participants provide urine, saliva and blood samples. Individuals also have anthropometric measurements, bio-impedance analysis, grip strength test, electrocardiogram (ECG), hepatobiliary ultrasonography, and oral examination (Table 2). Participants receive the results of their baseline exams (blood glucose, lipid and liver profile, blood pressure, nutritional evaluation, electrocardiogram and hepatobiliary ultrasound) and medical advice if any abnormality is detected, including reference to the emergency room if needed.

**Table 1:** Baseline questionnaire data collected in MAUCO 2014-2016

Factors	Instruments
Quality of life and lifestyle	General health^a^; Tobacco, alcohol, medications, oral health^b^
Cardiovascular diseases	Medical history^b,c^
Digestive diseases and cancer	Personal and family medical history and symptoms^d^
Respiratory diseases	Medical history^b^
Mental health	Depression^e^, medical history, cognitive status^f^, Trail making
	test^g^, stress^h^, instrumental activities of daily living^i^.
Nutritional status	Mediterranean diet^j^, cooking method of meals^k^
Physical condition	Physical activity^d^, sedentary behavior^l^
Hearing and seeing status	Medical history^b^
Socio-cultural risks	Socioeconomic^b^, social networks^m^, employment status^n^
Environmental & work exposures	Chemicals, pesticides^o^
Women’s reproductive health	Hormonal^b^, reproductive, cervical and breast cancer screening^d^.

^a^SF-12 Instrumental Activities of daily living questionnaire

^b^Encuestas Nacionales de Salud de Chile (ENS) (Chilean National Health Survey) 2009. http://www.dinta.cl/wp-dintacl/wp-content/uploads/Presentacion-ENSalud-2010.pdf

^c^Minnesota Living with Heart failure Questionnaire

^d^ENS 2003. http://www.ine.es/metodologia/t15/t1530419.htm

^e^Patient Health Questionnaire PHQ-9

^f^Addenbrooke’s cognitive examination- Revised, Spanish version: Muñoz-Neira et al. 2012 Rev Med Chilehilee 2012;140:1006-1013

^g^Reitan, C. (1992). The Trail Making Test: Manual for administration and scoring. Tucson: The Reitan Neuropsychological Laboratory

^h^Lanas et al. Rev Med Chile 2007 136: 555-560

^i^Muñoz-Neira et al. 2012 Dement Geriatr Cogn Disord 2012;33:361–371

^j^Leighton et al. Public Health Nutrition. 2009; 12(9A), 1635–1643

^k^PLCO Prostate, Lung, Colorectal and Ovarian Cancer Screening Trial

^l^GPAQ- WHO Global Physical Activity Questionnaire

^m^Sapaj et al. J Epidemiol Community Health. 2008;62(9):790–2

^n^Encuesta Nacional de Empleo, Trabajo, Calidad de Vida y Salud de los Trabajadores y Trabajadoras de Chile 2009-2010 (ENETS) (National Survey of Employment, Labour, Quality of Life and Health of the Workers of Chile 2009-2010) http://www.isl.gob.cl/wp-content/uploads/2011/10/PRESENTACION-ENETS-2009-2010-FINALINTERINSTITUCIONAL.pdf

^o^Prospective Investigation of Pesticide Applicators’ Health Study (PIPAH)

**Table 2:** Baseline physical examinations, imaging studies and laboratory tests in MAUCO 2014-2016

Health condition examined	Instruments & tests
Physical condition (frailty)	Peak expiratory flow (PEF), handgrip strength, bio-impedance, self-reported waking speed, Up and Go test 2.5 m
Cardiovascular system	Blood pressure and heart rate, electrocardiogram, blood lipids
Hepatobiliary system	Hepatobiliary ultrasound, liver profile (blood GOT, GPT, bilirubin)
Diabetes	Glycaemia
Pulmonary function	Peak expiratory flow (PEF)
Cognitive function	Attention, memory, language and executive functions. Activities of daily living.
Oral health	Tooth count, oral examination for periodontal disease
Anthropometric measurements	Height; weight; waist, hip, neck & ankle circumference; arm span
Basal inflammatory status	Immunological panel in blood^a^
Infectious disease	biomarkers for *H. pylori* ^a^ *, S. typhi* ^a^; microbiota characteristics in saliva^a^
Genetic determinants	DNA biobanking for future genomic studies
Environmental exposure	Aflatoxin biomarkers in urine and blood^a^, pesticides biomarkers in blood or urine^a^, metals in urine^a^, nitrates in urine and water^a^.

^a^Planed for the baseline assessment

### Time line: enrollment and follow-up

Funding for the project was granted in December 2013 and field work began in July 2014 with the census and a pilot project to develop strategies and instruments. Enrollment, including bio-sampling, began in December 2014 and is expected to be completed by December 2016. Follow-up visits consist of a shorter version of the baseline measurements to assess changes in exposure and health conditions. This evaluation will also include in-depth surveys of sub-groups, such us participants exposed to pesticides or diagnosed with gallstones. These visits will occur 2, 5 and 7 years after enrollment.

### Surveillance of health events and expected events

To identify all health events affecting the cohort participants, in particular serious events requiring hospitalization, we have established formal agreements with public hospitals in the region where health events are likely to take place. These agreements will permit us to link individuals who are entering a hospital with the MAUCO database, which will automatically send a report to the surveillance team.

Based on Chilean cancer surveillance [15], regional mortality and international incidence data, we expect 1,265 health events in the first 5 years of follow-up, including 385 cases of new-onset diabetes mellitus, 309 cases of cancer, 120 cases of acute myocardial infarction, 115 cases of chronic pulmonary disease, 160 cardiovascular events (cardiac failure and cerebral stroke) and 230 deaths.

### Follow-up and retention strategy

To improve participation rates and maintain high adherence to study protocols over time, a comprehensive retention strategy is being developed. The first qualitative study explored motivations, beliefs and preferences of MAUCO participants to adhere to the study. This information is being integrated in the development of a retention strategy under the principles of Community-Based Participatory Research (CBPR) [17], which aims to engage the Molina community as a partner in the study, promoting a good understanding of study goals and local ownership in the study by community members. This will also contribute to the design and implementation of locally appropriate initiatives oriented towards chronic disease prevention.

### Maule Cohort Bio-Bank

MAUCO biological samples are stored following the US National Cancer Institute (NCI), Biobank Quality Control regulation [18]. Standard operating procedures are in place to ensure the quality of the MAUCO biological samples from collection to storage. From the MAUCO station, samples are transported at 4 °C to the near-by laboratory in the Molina hospital. Samples are processed within 2 h, and 30 to 50 aliquots per subject are stored at -80 °C in the MAUCO Bio-Bank at the Molina hospital; these samples are periodically transferred to the Central Bio-Bank in the Pathology Department of the Pontificia Universidad Católica de Chile in Santiago for long-term storage at -80 °C. Forms, samples, and aliquots have unique barcodes that are linked to a sample tracking system. An inventory system of biological samples is used to identify the location and available quantity of each aliquot by Biological Specimen Inventory (BSI) software (www.bsisystems.com).

### MAUCO Data Bank

The MAUCO information system ensures safe, complete and accurate data flow including data collection, data entry and generation of databases. Data collection, supervised by the field coordinator, is conducted by health technicians using printed questionnaires. Data entry, supervised by the data-manager, is done locally using two services for data entry and dataflow: REDCap™ (Research Electronic Data Capture) [19] and ProcessMaker® (http://www.processmaker.com). REDCap™ is a secure, web-based application designed to support data capture for research studies, while ProcessMaker® is the study management system that facilitates processes and procedures. The management databases system MySQL (https://www.mysql.com) is used to connect the online servers with double backup (cloud and local).

### Data analysis plan

MAUCO’s Data-Bank (MDB) includes individuals` baseline data, follow-up data, surveillance findings and the results of any other measurement carried out as part of a sub-study. The cohort study design allows for a broad range of hypothesis to be tested longitudinally. Examples of initial planned analyses include the association of CDs with: 1) pesticides and mycotoxins, 2) labor fragility and social and psychosocial factors and 3) inflammatory conditions such as gallstones. Our hypothesis is that CDs share risk factors leading to chronic inflammation and oxidative stress as the common underlying mechanism for the development of cancer, cardiovascular disease and other chronic conditions [20–23].

### Ethics

The study protocol was approved by ethics committees at Pontificia Universidad Católica de Chile and the Maule Regional Service of the Chilean Ministry of Health. Eligible individuals are invited to voluntarily participate in the study, and the consent form has to be fully read and explained to the subjects. Participants´ privacy is protected throughout the study process. Individuals with abnormal results are informed and referred to their health care system accordingly.

### Regulation of access to data/biospecimens

The access to data and biospecimens is regulated by the ACCDiS directorate in accordance with the local Institutional Review Board authorization. The regulatory system was developed following the UK Centre for Longitudinal Studies [24] protocol, and aim to make the data and biospecimens available to other researchers while ensuring that ethical and scientific principles are fulfilled. All data requests must be approved by the Advisory Board and a bilateral agreement must be signed before sharing of data. International data requests can be allowed following the same algorithm, using data views. Access to the server will not be granted.

### Outreach/dissemination

Outreach is targeted to the Molina community to inform study findings and promote participation over time. We developed a MAUCO logo, typography and printed material (color brochures or leaflets) with information about the study and its field team and interviews and short capsules for the local radios, TV and newspapers, including the municipal newsletter and the Molina hospital online newsletter. The MAUCO website is currently in its beta version. Broader dissemination has mainly been channeled through regional and national print media (newspapers, magazines) featuring news reports and interviews.

## Main strengths and weaknesses of the study

Among the strengths we can mention that this study is conducted in a county with a particularly high burden of CDs and unique exposures, where all citizens aged 38 to 74 years are invited to participate. The county economy is agriculture based, including large vineyards. Molina inhabitants are widely exposed to pesticides, mycotoxins and alcohol consumption, which make this study particularly well-suited for assessing the role of these environmental factors in the development of chronic diseases. We expect 3,000 subjects will be agricultural workers. In fact MAUCO is part of the WHO AGRICOH [25], an agricultural cohort consortium, along with 27 other cohorts from the rest of the world [26]. MAUCO is the first population-based CD cohort in Chile of this magnitude, with such a broad spectrum of exposures and health outcomes. A particular novelty is that MAUCO was developed through the interdisciplinary collaboration of epidemiologists with basic and clinical scientists. Data and biological samples from the population study will inform the basic and clinical scientists, while insights from laboratory experiments will help generate new, more specific hypotheses at the population level. Researchers at ACCDiS are national and international leaders in their areas and the MAUCO team has great experience in large population-based studies.

Local and regional authorities’ and institutions (Molina´s Municipality and Hospital, Maule Health Service, Universidad Católica del Maule), CONICYT, and the primary Grantee Universities (Universdad de Chile and Pontificia Universidad Católica de Chile) are highly committed to the project, securing sustainability of the cohort.

This project has a 10-year institutional funding commitment, with the possibility of re-applying for another 10-year period. Principal and associate investigators and collaborators are applying for additional sources of funding. As an example the US National Cancer Institute of the National Institutes of Health has provided guidance and training to help establish the MAUCO Bio-Bank and equipment to facilitate a sub-cohort of individuals with gallstones.

The study’s main challenges are the lack of experience in Chile with population-based prospective studies with long-term products, requiring large amounts of resources (financial, human, infrastructure) and complex organization to secure sustainability and high quality. Also, sample and data storage is a developing field in Chile, MAUCO´s Bio-Bank is one of the first initiatives of this magnitude. Another limitation is related to the external validity of our findings. MAUCO represents agricultural populations from small cities in Latin America. Some results may not be applicable to residents of large urban areas.

## Discussion

MAUCO will provide high quality data and biological samples that will be a resource for national and international research. Prevalence and dynamics of risk factors for CDs and their biomarkers will be measured in a unique epidemiological setting thus far not considered in the large cohorts of developed countries. This cohort is similar in aims to European cohorts, such as each of the German National Cohorts [27] and LifeLines Cohort Study & Biobank [28]. MAUCO is exploring a broad range of exposures from genetics to environmental exposures and their impact on health status, quality of life, aging and cognitive performance. After an 8-year embargo to complete the baseline measurements, data and samples will be available for the scientific community. MAUCO will permit assessing the effect of common risk factors and establishing the reference values in this population, providing key data for the design of interventions suitable for this group. Furthermore, MAUCO will provide the strategies, methods and tools to conduct this type of study in other populations with diverse epidemiological settings.

## Ethics approval

The study protocol was approved by Ethics Committees at Pontificia Universidad Católica de Chile (N° 14-141) and the Maule Regional Service of the Chilean Ministry of Health.

## Consent

An informed consent to participate in the study is obtained from all participants of MAUCO.
